# Expressive writing intervention and self-reported physical health out-comes – Results from a nationwide randomized controlled trial with breast cancer patients

**DOI:** 10.1371/journal.pone.0192729

**Published:** 2018-02-23

**Authors:** Mikael B. Jensen-Johansen, Mia S. O’Toole, Søren Christensen, Heiddis Valdimarsdottir, Sandra Zakowski, Dana H. Bovbjerg, Anders B. Jensen, Robert Zachariae

**Affiliations:** 1 VIA University College, Aarhus, Denmark; 2 Unit for Psychooncology and Health Psychology, Department of Oncology, Aarhus University Hospital, and Department of Psychology and Behavioural Science, University of Aarhus, Aarhus, Denmark; 3 Reykjavik University, Reykjavik, Iceland; 4 Mount Sinai School of Medicine, New York, United States of America; 5 Illinois School of Professional Psychology, Argosy University, Chicago, United States of America; 6 Biobehavioral Oncology Program, UPMC Hillman Cancer Center, Pittsburgh, PA, United States of America; 7 Departments of: Psychiatry, Psychology, Behavioral Community Health Sciences and Health & Community Systems, University of Pittsburgh, Pittsburgh, PA, United States of America; 8 Department of Oncology, Aarhus University Hospital, Aarhus, Denmark; Karolinska Institutet, SWEDEN

## Abstract

The objective was to examine the effect of Expressive Writing Intervention (EWI) on self-reported physical symptoms and healthcare utilization in a nationwide randomized controlled trial with Danish women treated for primary breast cancer, and to explore participant characteristics related to emotion regulation as possible moderators of the effect. Women who had recently completed treatment for primary breast cancer (n = 507) were randomly assigned to three 20 min. home-based writing exercises, one week apart, focusing on emotional disclosure (EWI) of a distressing experience (their cancer or a non-cancer topic) or a non-disclosing topic (control). Outcomes were self-reported physical symptoms and healthcare utilization (visits and telephone contacts with GP) 3 and 9 months post-intervention. Potential moderators were repressive coping, alexithymia, rumination, social constraints, and writing topic. Results revealed no group by time interaction effects for any outcomes. Moderation analyses showed that 1) low alexithymic women in the EWI group showed larger decreases in GP telephone calls over time than both high alexithymic women and controls and 2) women in the EWI group writing about their own cancer, but not women writing about other topics, showed a larger decrease than controls. The results from this large randomized trial are concordant with previous findings showing that EWI is unlikely to be a generally applicable intervention to improve health-related outcomes in cancer patients and cancer survivors. However, written disclosure might have a beneficial impact for individuals who write about their own cancer, as well as for those low in alexithymia.

## Introduction

The ability to express and regulate emotions is thought to play an important role in promoting and maintaining physical health [1]. Emotion dysregulation in the form of disattention to signals of distress has been hypothesized to result in *physiological* dysregulation due to cortical overriding of the autonomic nervous system and immunoregulatory homeodynamic mechanisms as well as increased risk-related behaviors [2]. Although the evidence is mixed [3], various measures of emotional avoidance and repressive coping have been found associated with both development [4, 5], and poor prognosis of breast cancer [6–8]. While acknowledging the methodological challenges in differentiating and measuring such concepts of impaired emotional processing [9], it remains relevant to explore possible effects of interventions aimed at improving emotion regulation on physical health outcomes in cancer patients and survivors. Expressive Writing Intervention (EWI)], is one type of intervention aimed at improving emotion regulation through emotional disclosure, and the present study extends our previously published findings concerning effects of EWI on psychological distress in our large population-based, randomized, controlled trial of EWI with women treated for primary breast cancer [11] by analyzing the effects on physical health outcomes.

According to the EWI paradigm, it may be possible to improve both mental and physical health through improving *self-regulation* of *emotion-related* experiences [12], and the results of early studies by Pennebaker and colleagues [13–15], did indeed suggest that EWI, instructing participants to write as little as 20 min for 3–4 days about emotions associated with a traumatic event, may be associated with psychological benefits as well as improvements in a number of physical health-related factors, including immune changes, reduced physical symptoms, and reduced health care utilization. Later studies have provided additional support for beneficial effects in both healthy samples [16, 17], and samples with physical illness [18–20]. The most comprehensive meta-analysis published so far [21] reviewed 146 randomized, controlled trials (RCTs) of various types of experimental disclosure in healthy and clinical samples and found statistically significant, but small, overall effect (r = 0.075≈Cohen’s *d =* 0.15). Specifically reviewing EWI with cancer patients and survivors, a recent meta-analysis of 16 RCTs suggests a negligible overall effect (Hedges’s *g* = 0.04;*p* = 0.38) [22]. Most pertinent to the present study are the 11 studies which investigated effects of EWI on various physical health outcomes in cancer patients, revealing a small and statistically non-significant effect (*g* = 0.08) [22].

Taken together, the research on physical health outcomes is heterogeneous and relatively sparse. However, results for both cancer and other populations suggest that written emotional disclosure may be more relevant for some participants than others. For instance, an emotionally under-regulated person tends to have little control over responses to provoking stimuli, and emotions are thus experienced more intensely with subsequent exaggerated physiological arousal and increased risk of, e.g. cardiovascular disorders [12]. An overregulated individual, in contrast, tends to avoid, inhibit, or suppress emotional responses, which may require physiological work that facilitates sympathetic dysregulation [23] with detrimental effects, e.g. on the immune function [20]. From an emotion regulation perspective, the following factors directly or indirectly associated with impaired emotion processing could thus be viewed as potential moderators. *Repressive coping* is defined as a tendency to under-report negative affect as a result of non-conscious protection of their self-esteem [24, 25], which may interfere with the effects of written disclosure [26]. *Alexithymia* is an insufficient ability to process emotions through cognitive mechanisms, and individuals who have difficulties describing their emotions, or lack the skills and ability to reflect upon and process their experiences, may benefit less from EWI [26, 27]. In contrast, *rumination* is viewed as conscious, spontaneous, and repetitive thoughts concerning past negative information that may influence affective responses to negative events [28]. Tendencies to ruminate have been associated with poorer self-reported physical health and increased health care utilization [29]. Although studies suggest that reduced rumination may explain the beneficial effect of EWI [30, 31], rumination has not yet been studied as a potential moderator of EWI in cancer patients. From a social perspective, *social constraints*, i.e., perceived inadequacy of social support resulting in a reluctance to express thoughts and feelings about a specific stressor [32], may hinder people from confronting and processing traumatic experiences, e.g., cancer [33], resulting in disturbed illness adaptation [34]. EWI could buffer against the negative consequences of social constraints by providing an opportunity to compensate for the lack of social support. This hypothesis has indeed found some support in EWI studies with cancer patients [22]. Finally, the *writing topic* in EWI studies with cancer patients has generally been constrained to their cancer, and it is unclear whether effects of EWI may differ according to the participants´ choice of writing topic. Hypothetically, some individuals may experience writing about their own cancer as threatening and therefore choose to write about more superficial topics or less specific traumas, thereby perhaps reducing the possibility of an effect. It might on the other hand also be beneficial for some patients not to dwell on concerns about their breast cancer, as they may be unable to cope efficiently with highly emotional thoughts and negative emotions within the short time-frame of EWI.

Taken together, there continues to be a need of larger, well-controlled trials evaluating: a) whether there are any relevant and meaningful main effects of EWI on physical health-related outcomes and, in particular, b) if any moderating effects can be found which could inform the selection of patients who could potentially benefit from the intervention. As a part of a large, nationwide EWI intervention (11), we therefore examined the effect of EWI on physical health-related outcomes in a nationwide sample of Danish women treated for early-stage breast cancer. First, we tested the hypotheses that women assigned to EWI would experience improvements in *self-reported physical symptoms* and *reduced health care utilization* (self-reported visits and telephone calls to the General Practitioner) compared to controls writing about a neutral topic. Secondly, we explored the possible *moderating effects* of the following participant characteristics: repressive coping, alexithymia, rumination, social constraints, and choice of writing topic.

## Materials and methods

A nationwide, randomized controlled trial was conducted in collaboration with the Danish Breast Cancer Cooperative Group (DBCG) and 14 out of the 16 surgical departments responsible for treating breast cancer in Denmark. Detailed information regarding methods has been described in a previous report of the effects of EWI on psychological health outcomes in the present sample [11].

### Participants

**Fig 1:**
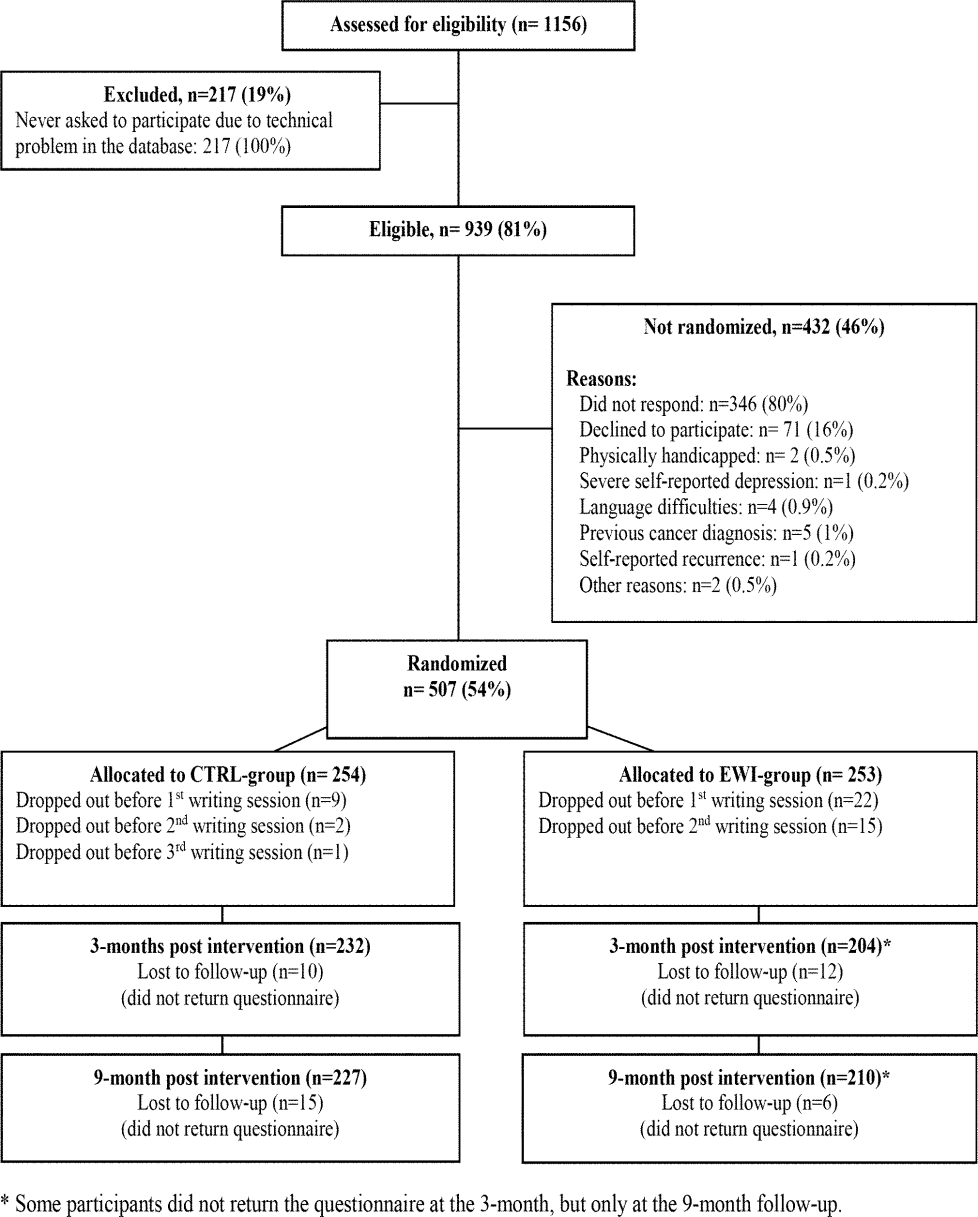
CONSORT study flow diagram.

Participants considered for eligibility were 1156 women, able to read and write Danish, aged 18–70 years, who had been treated surgically (mastectomy or lumpectomy) over a 6-month period within 3 weeks of a diagnosis for invasive breast cancer stage I and II, and subsequently receiving adjuvant treatment according to Danish national guidelines [35, 36]. As shown in Fig 1, ineligibility and non-response resulted in the randomization of 507 women (age: 27–70 years) to either the EWI and or control (CTRL) group. The study was approved by The Central Denmark Region Committee on Health Research Ethics and the Danish Data Protection Agency.

### Procedure

The present study presents the results for physical health outcomes from our nationwide EWI intervention study, for which the overall procedures have been described in detail elsewhere [11]. In brief, the women were contacted by mail between 8 and 12 weeks after surgery, or if indicated in the treatment protocol, 4 weeks after completion of chemotherapy and/or radiotherapy. After having given informed consent and completed the baseline questionnaire, the women were randomized to EWI (N = 253) or CTRL (N = 254) using a computerized stratified sampling method with four mutually exclusive strata reflecting the four standard adjuvant cancer treatment protocols (chemotherapy, radiotherapy, both, or none). The home-based writing instructions followed the original procedure developed by Pennebaker and colleagues [10], with trained research assistant instructing both groups by telephone to write for 20 min, once a week, over a 3-week period. On the first writing day, participants received a longer introduction to the writing exercises and were informed that they might experience changes in their mood. On each writing day, research assistants contacted the participants by telephone to initiate the writing session and contacted them again after 20 min to terminate the writing. At both time points, mood questionnaires were filled out. The participants were asked to write about a traumatic or distressing event and to explore their deepest feelings and emotions associated with this experience.

They were free to write about their breast cancer as well as non-cancer experiences and to switch topics during the intervention. The CTRL group participants were asked to write as objectively and detailed as possible in an emotionally neutral manner about their daily activities. Participants were mailed follow-up questionnaires 3 and 9 months post-intervention with a single reminder after 2 weeks. The trial hypotheses and group assignments were masked by naming the project the ‘Stress-Management & Quality-of-Life Brief Writing Exercise Program’.

### Measures

#### Background data

Treatment and other clinical variables obtained from the DBCG registry [37] included: age, menopausal status, type of treatment (surgery, chemotherapy, or radiotherapy), tumor size, lymph node status, estrogen receptor status, and grade of anaplasia. Sociodemographic data included age and marital status.

#### Health-related outcomes

Physical symptoms were assessed with the 15-item Patient Health Questionnaire Somatic Symptoms subscale (PHQ-15) [38]. Healthcare utilization was measured with two questions about the number of health care visits (GP-Visits) and telephone calls (GP-Phone) to their general practitioner (GP) at baseline (since surgery), and at the follow-ups (since the intervention). These variables were transformed to reflect contacts per month.

#### Moderators

Repressive coping style was, as suggested by Weinberger [24] assessed with the combined Marlowe-Crowne Social Desirability (MCSD) and Taylor Manifest Anxiety Scale (TMAS-Bendig Short Form) (MCSD-TMAS) [39, 40]. Alexithymia was measured with The Toronto Alexithymia Scale (TAS-20) [41]. The TAS-20 consists of three subscales measuring *Difficulties Identifying Feelings* (TAS-DIF), *Difficulties Describing Feelings* (TAS-DDF), *External Oriented Thinking* (TAS-EOT) and a total score (TAS-TOTAL). Rumination was assessed with the Emotional Control Questionnaire (Rehearsal Subscale) (ECQ-R) [42]. The possible constraining effects of the social environment on attempts to talk about breast cancer were assessed with the Social Constraints Scale (SCS-C) [32]. Writing topic was registered after each session. If the women reported that they had written “*about emotions related to my own cancer or treatment*” in one or more sessions, they were considered belonging to the “*own cancer*” writing group. If they consistently had written about “*emotions concerning traumatic topics other than my own cancer*”, they were considered belonging to the “*other topics*” writing group.

#### Covariates

Due to uneven number of dropouts in the EWI and control groups and dropout being associated with age and depressive symptoms, age and depressive symptoms at baseline were included as covariates in a second set of analyses. Depressive symptoms were measured with the short 13-item version of Beck Depression Inventory (BDI-SF) [43, 44].

### Reliability

The Danish versions of the questionnaires used in the present study had previously demonstrated acceptable internal consistencies (Cronbach’s alpha: 0.63–0.90) in samples of cancer patients [25, 36] and healthy elderly people [45]. Consistency between an independent rater (the first author, MBJ-J) and the self-reported EWI and CTRL writing topics was assessed for a subsample of 237 essays written by 49 EWI and 30 CTRL participants (20% of complete cases). Inter-rater reliability was 0.75 (Kappa statistic, p < 0.001).

### Manipulation check

As a manipulation check, all participants were asked questions about their writing process, and their emotional responses to the writing were assessed by phone immediately after each writing session with a 5-item version of the Profile of Mood States (POMS) [46] and three items from a Passive Positive Mood Scale (PPMS) developed for the present study [11].

### Statistical power

The study was designed to approach 1250 eligible patients over a 6-month period. Estimating a 50% inclusion rate and a 20% dropout rate between baseline and follow-up, we aimed to include 2 x 250 participants with complete data at 3 months follow-up. This would provide a statistical power of 80.1% to detect a statistically significant result (p<0.05, two-tailed) corresponding to a small effect size (Cohen’s *d*) of 0.25, comparable to the overall weighted effect size (0.22) for the combined psychological and physical health outcomes reported in comparable EWI studies of cancer patients available at the time when planning the study [47–50].

### Statistical analysis

As recommended [51], subscale totals with >50% missing item values were coded as missing. If missing item values were ≤50%, they were substituted with the mean of the remaining completed items on the subscale for each subject.

Data were analyzed as intention-to-treat with mixed linear models (MLMs) comparing groups over time. This approach, using all available data without any ad hoc imputation of missing observations is more powerful than other options for handling missing observations in longitudinal clinical trials with missing values [52]. Thus, data were neither trimmed nor imputed, rather, all available data. points informed the analyses. The data were hierarchically arranged in two levels. Level-1 variables referred to the repeated measures that were nested within individuals at level 2. Two sets of models were evaluated. One set included the group variable EWI vs. CTRL. The group variable in the other set of analyses included three groups: EWI participants writing about their own cancer; EWI participants writing about a topic other than their own cancer; and the neutral writing CTRL group. The time variable was treated as continuous, reflecting the measuring points in months. Due to skewness, a log-transformed variable was used for physical symptoms, and the model estimated with the maximum likelihood method. As GP visits and telephone calls were “count data”, we used a multilevel negative binomial model [53]. Parameter statistics were converted into Cohen’s *d*, where 0.2, 0.5, and 0.8 were considered a small, medium, and large effect sizes, respectively [54]. All analyses were performed in Stata version 14 and SPSS version 24.

## Results

### Participants and attrition

Of the 507 women randomized, 31 women cancelled their participation before or during the 1^st^ writing session, and 18 dropped out during the 3- week intervention period. Another 22 and 21 women did not return questionnaires at 3 and 9 months (See Fig 1). The 87 (17.2%) dropouts were older and more likely to be post-menopausal than participants. EWI dropouts were older and had more depressive symptoms [11] than CTRL dropouts. Age and depressive symptoms were therefore included in the analyses. Conducting the analyses with and without these covariates did not substantially change the results.

### Baseline data and intervention characteristics

No baseline differences between EWI and CTRL were found for the demographic, disease, treatment, outcome or moderator variables analyzed in the present study (See Table 1).

**Table 1:** Descriptive statistics and baseline differences.

Variables	EWI group		CTRL group		
	N	M (SD)	N	M (SD)	*p*
Sample size (N)	253	-	254	-	-
Age	253	53.6 (9.0)	254	53.6 (9.2)	0.994
Married/cohabiting (%)	202	80%	205	81%	0.806
DBCG treatment protocols *					
A (%)	62	25%	58	23%	0.658
B (%)	68	27%	79	31%	0.295
C (%)	68	27%	73	29%	0.640
D (%)	55	22%	44	17%	0.210
Nodal status (≥1) (%)	104	41%	113	45%	0.442
Mastectomy (%)	96	38%	99	39%	0.811
Tumorectory (%)	157	64%	155	61%	0.811
Radiotherapy (%)	195	77%	197	78%	0.897
**Physical Symptoms**					
PHQ T1	249	6.81 (4.02)	252	6.40 (4.03)	0.108
PHQ T2	201	7.00 (3.88)	226	6.62 (4.05)	-
PHQ T3	208	7.08 (4.11)	223	6.72 (4.33)	-
**General Practitioner (GP) visits**					
GP visit T1	202	2.08 (3.2)	223	2.03 (2.5)	0.849
GP visit T2	186	1.61 (1.21)	205	1.66 (1.83)	-
GP visit T3	188	1.21 (1.61)	204	1.32 (1.35)	-
**General Practitioner (GP) telephone calls**					
GP-phone T1	204	1.12 (1.71)	217	1.23 (1.98)	0.551
GP-phone T2	186	0.89 (1.61)	206	0.86 (1.37)	-
GP-phone T3	187	0.80 (1.27)	203	0.95 (1.31)	-

*) Treatment protocols: A: -CEF, _Tam, _RT [ER+ and Ts<20 mm and (Grade I or non-ductal) & N0 & age ≥35 year]; B: +CEF, +Tam [(ER+ or ER unknown) and pre-menopausal]; C: _CEF, +Tam (ER+ and post-menopausal); D: +CEF, _Tam [ER_ and (pre-menopausal or post-menopausal)]. Abbreviations: ER/PR+/_, estrogen/progesterone receptor status positive/negative; N0/≥1, lymph node status negative/positive; Ts. tumor size; RT, radiotherapy; CEF, cyclophosphamide, epirubicin, and fluorouracil; TAM, tamoxifen.

All participants wrote for 3×20 min. Women allocated to the EWI group spent slightly longer time in total on the telephone with research assistants (Mean: 35.3 min/session) than the CTRL group (31.7 min.) (p<0.001). No further between-group differences were found for intervention characteristics, including number of minutes spent on the actual writings and number of days between baseline and intervention, between the first and last writing session, or between the last writing session and completion of follow-up questionnaires. No statistically significant differences were found in the frequency of EWI and CTRL sessions led by the 12 research assistants..

### Writing topic group characteristics

Of the EWI participants, who returned all three essays, 108 chose to write about their own cancer at least once and 85 about other traumatic experiences, e.g., physical abuse, death of a family member, and divorce. Women who had received a mastectomy, had received chemotherapy, had used pain medication, or received professional help were more likely to write about having breast cancer than other traumas (chi2 tests; *p*s = 0.012–0.026).

### Manipulation check

Significant mood changes (p<0.001) in the expected directions were found immediately after the sessions, i.e., increased negative and reduced positive mood in EWI compared to CTRL. Effect sizes (Cohen’s *d*) were large (0.84–1.04). For further details, see [11].

### Physical symptoms and health care utilization

As shown in Tables 1 and 2, medium to large and statistically significant effects of time were found for number of GP visits and GP telephone calls, but not physical symptoms. No Time×Group interaction effects reached statistical significance. See descriptive data in Table 1, and results in Table 2A.

**Table 2:** Results from MLMs (baseline, 3- and 9 month follow-up) (Time × Group) of physical symptoms (PHQ), number of GP visits, and number of GP telephone contacts (2a), and results of moderation analyses with repressive coping (2b), social constraints (SCS-C) (2bc), rumination (ECQ) (2d), alexithymia (TAS-20) (2e), and writing topic (own cancer vs. other topic vs. neutral writing) included as moderators.

	EWI vs. CTRL		
	Effect		
	B (SE)	*p*	*d*
**2a) Physical symptoms (PHQ)**			
Time	0.03 (< .01)	.291	0.09
Time x Group	<0.01 (.01)	.854	0.02
**GP visits**			
Time	**-0.05** (.01)	**< .001**^**a**^	**0.65**
Time x Group	0.01 (.01)	.363	0.09
**GP telephone calls**			
Time	**-0.03** (.01)	**.006**^**a**^	**0.27**
Time x Group	0.01 (.02)	.543	0.06
**2b) Repressive Coping**			
**Time x Group x Repression**			
PHQ	-0.02 (.01)	.055	0.17
GP-visit	0.06 (.03)	.090	0.16
GP-phone	-0.01 (.05)	.806	0.03
**2c) Social Constraints (SCS)**			
**Time x Group x SCS**			
PHQ	<-0.01 (< .01)	.869	0.01
GP visit	<0.01 (< .01)	.257	0.10
GP-phone	<0.01 (< .01)	.676	0.04
**2d) Rumination (ECQ)**			
**Time x Group x ECQ**			
PHQ	<0.01 (< .01)	.927	0.03
GP-visit	<0.01 (< .01)	.575	0.05
GP-phone	<-0.01 (< .01)	.537	0.06
**2e) Alexithymia (TAS-20)**			
**Time x Group x TAS-total**			
PHQ	<-0.01 (< .01)	.515	0.06
GP-visit	<0.01 (< .01)	.657	0.04
GP-phone	<-0.01 (< .01)	**.029**^**b**^	**0.17**
**2f) Writing topic**			
**Own cancer vs. other topic vs. CTRL**	**χ2**	***p***	***d***
PHQ	2.84	.242	0.15
GP-visit	**8.90**	**.012**^**a**^	**0.30**
GP-phone	**13.89**	**.001**^**a**^	**0.37**

*Notes*: MLM: Multilevel Linear Models; *d*: Cohen’s *d*; a = Continued to be statistically significant (p<0.05) when including age and depressive symptoms (BDI-SF) in the model as covariates; b = Statistically significant (p<0.05) only when included, without covariates the result was trend-wise significant (p = 0.052), B(SE) = unstandardized parameter in the MLMs and its standard error; χ2 = chi square test for between-group differences over time.

### Repressive coping, social constraints, and rumination as moderators

Using the median splits of the MCSD (median = 22) and TMAS (median = 5), 31.8% of the women met the criteria for repressive coping style (defensive low anxious). The remaining three groups were collapsed, and the dichotomized variable (repressors, non-repressors) was used as moderator in the analyses. The possible moderators of social constraints (SCS) and rumination (ECQ) were entered as continuous variables. No significant interactions (Time×Group×moderator) were found (Table 2B, 2C and 2D), although repressive coping moderated the treatment effect at the trend-level for both PHQ and GP visits. For PHQ, the largest treatment effect difference was found for repressors, where repressors in the control group experienced the largest increase over time (p = 0.056). Concerning GP visits, the largest treatment effect difference was detected for non-repressors (p = 0.094), where the largest decrease was found for non-repressors in the EWI group.

### Alexithymia as moderator

**Fig 2:**
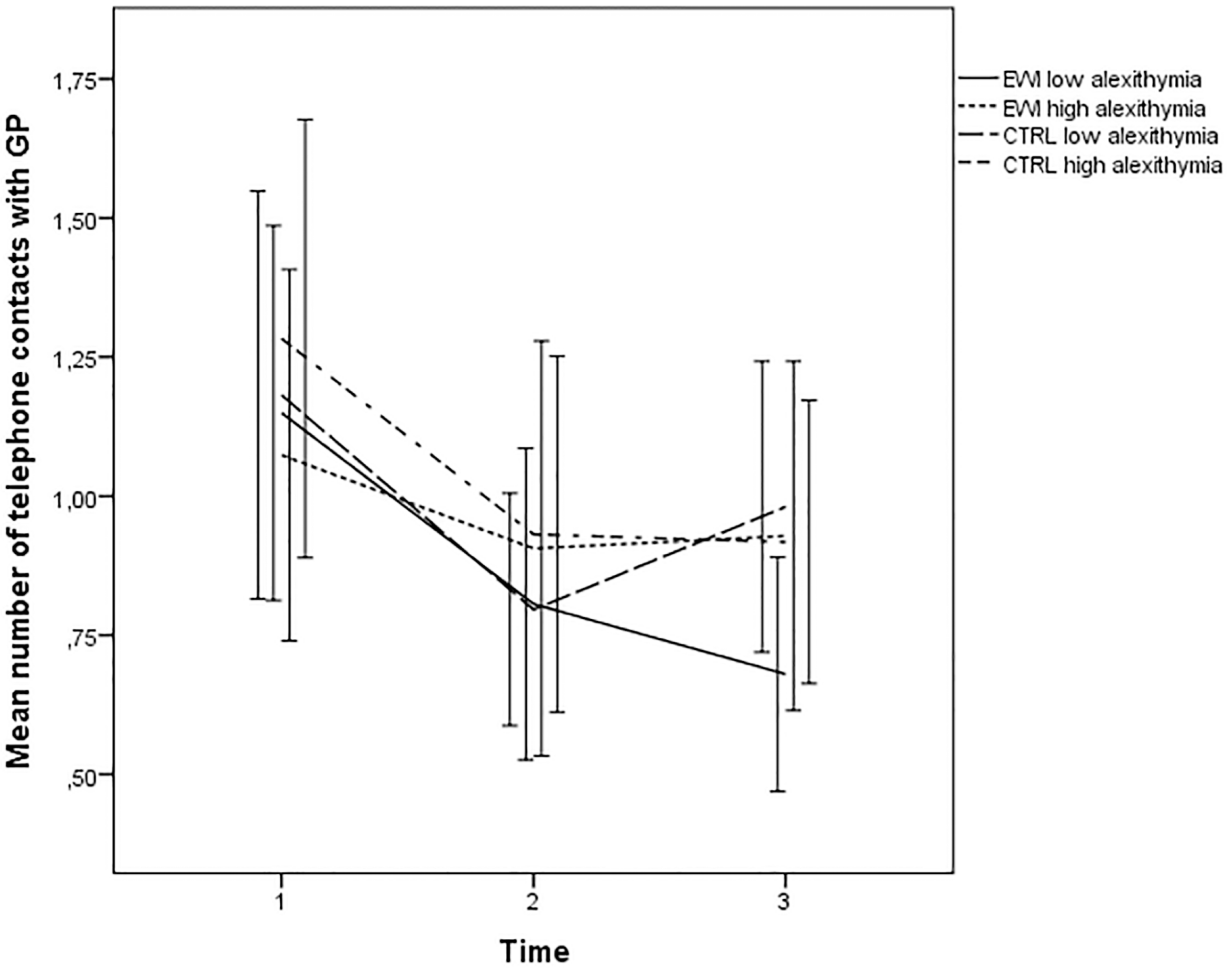
Alexithymia as moderator: Mean number of telephone contacts with general practitioner (GP) (Error bars: 95%CI) from baseline to follow-up of high and low alexithymic women in the EWI and neutral writing control group.

When exploring total alexithymia scores (TAS-20) as moderator, a statistically significant interaction (Time×Group×TAS-20) of small magnitude (0.17) was found for GP telephone calls, when adjusting for depressive symptoms and age (p = 0.029). Without these control variables, the results only showed a trend (p = 0.052). While alexithymia was not significantly associated with the outcome in the CTRL group, low alexithymic women in the EWI group showed larger decreases in GP telephone calls over time than high alexithymic women and controls (Table 2E and Fig 2). This effect appeared to be driven mainly by difficulties describing feeling (TAS-DDF subscale; p = 0.043) and externally oriented thinking (TAS-EOT subscale, p = 0.085), but not difficulties identifying feelings (TAS-DIF subscale, p = 0.412).

### Writing topic as moderator

**Fig 3:**
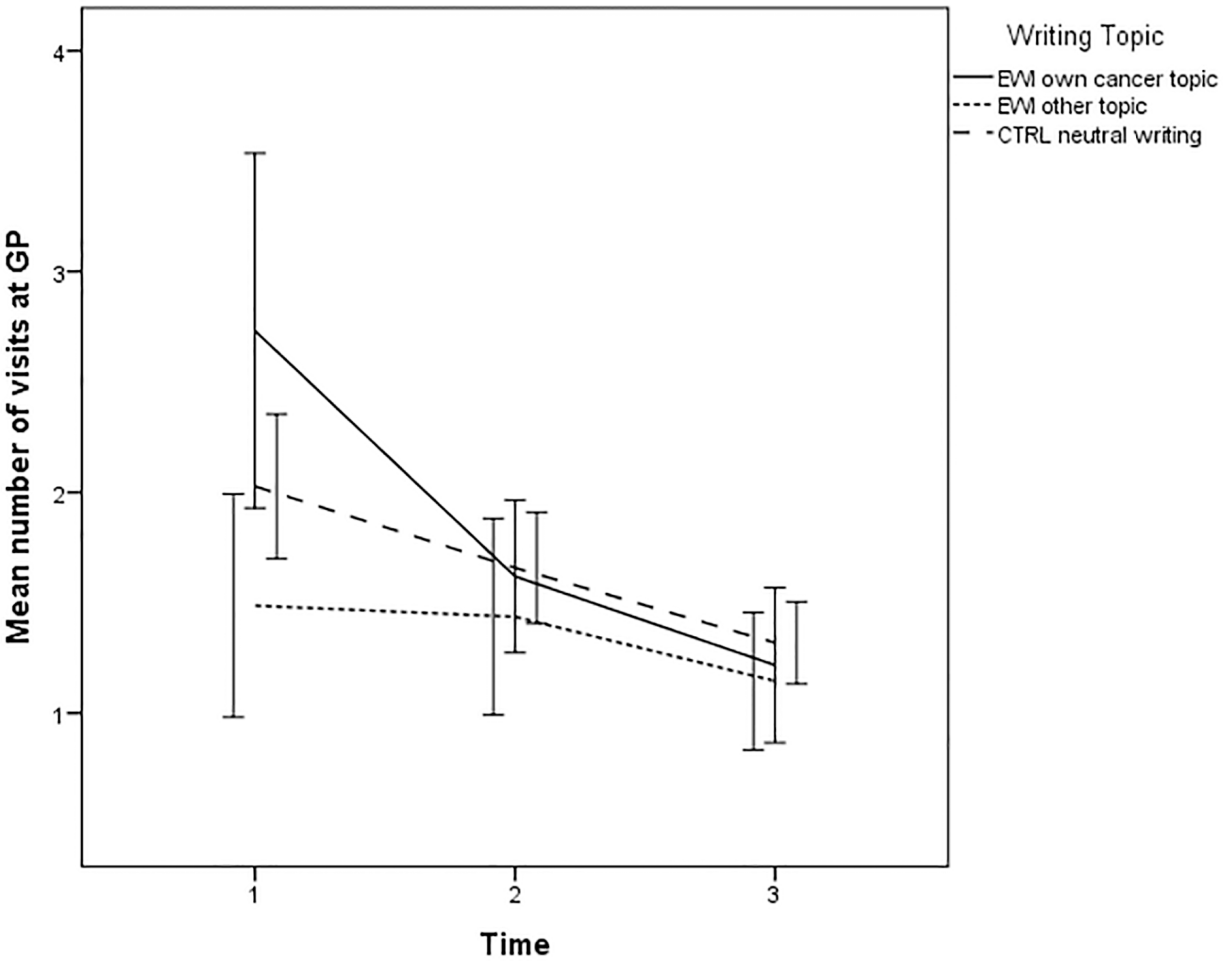
Writing topic as moderator: Mean number of visits to general practitioner (GP) (Error bars: 95%CI) from baseline to follow-up of EWI participants writing about their own cancer, EWI participants writing about other topics, and neutral writing controls.

**Fig 4:**
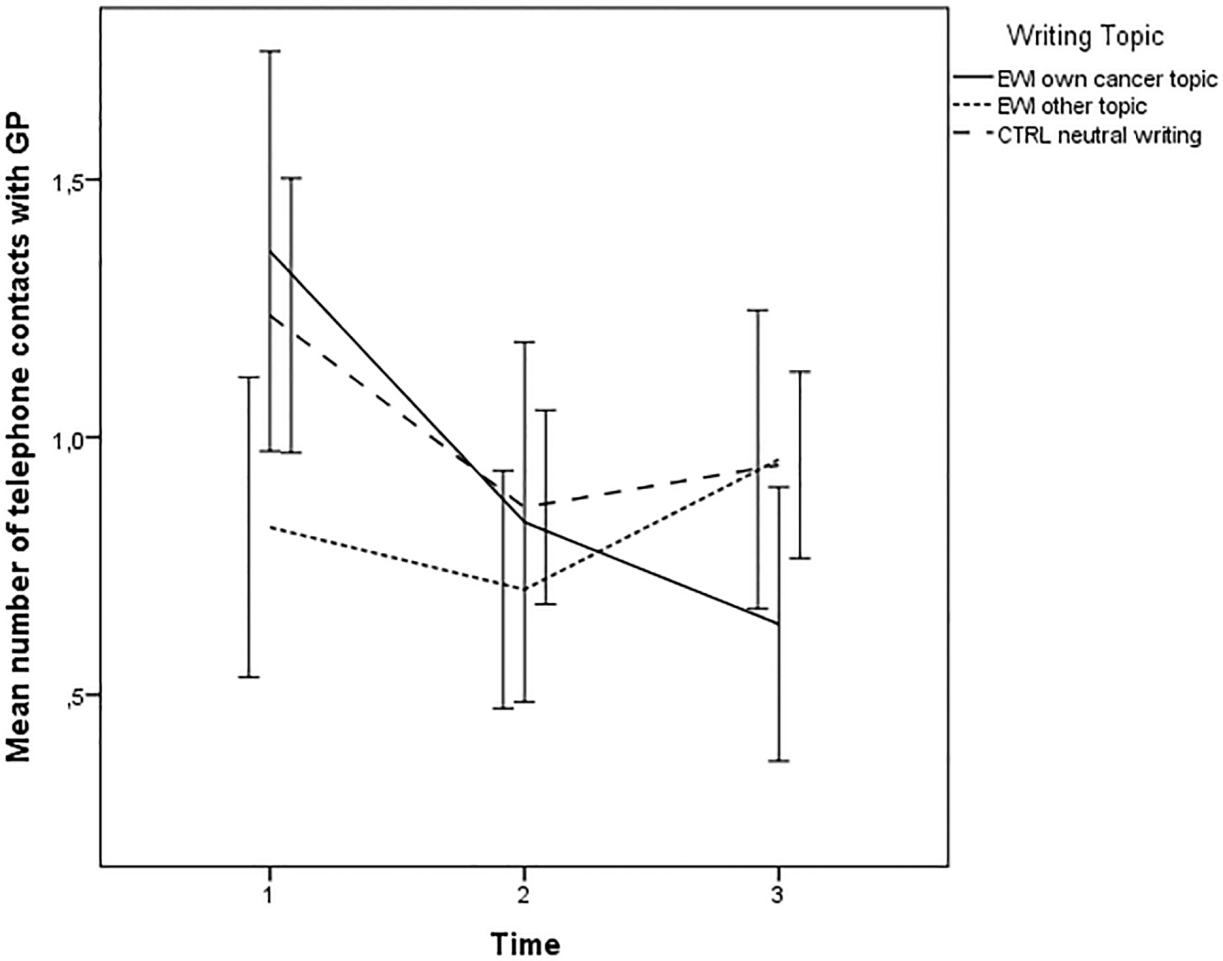
Writing topic as moderator: Mean number of telephone contacts with general practitioner (GP) (Error bars: 95%CI) from baseline to follow-up of EWI participants writing about their own cancer, EWI participants writing about other topics, and neutral writing controls.

Examining writing topic, time and their product as predictors of GP visits revealed a small, but significant, interaction effect (time×writing topic) (*p* = 0.012). Women writing about their own cancer (z = 2.40;*p* = 0.017), but not women writing about other topics (z = -0.97;*p* = 0.333) showed a larger decrease than CTRLs. The same pattern was detected for number of telephone contacts with GP (p = 0.001), where women writing about their own cancer (z = 2.56;*p* = 0.011), but not women writing about other topics (z = -1.89;*p* = 0.059), although trending, showed a larger decrease than CTRLs (Table 2F and Figs 3 and 4). The results remained statistically significant when entering age and depressive symptoms at baseline as covariates.

## Discussion

Our main hypotheses that EWI would lead to reductions in physical symptoms (*d* = 0.02) and reduced health care utilization in the form of GP visits (*d* = 0.09) and telephone contacts (*d* = 0.06) were not supported. Our results are thus concordant with the overall results (*g* = 0.08) of a meta-analysis of 11 previous EWI studies on physical health-related outcomes, suggesting that EWI is ineffective for cancer patients and survivors [22].

Our results were also in line with the notion that EWI could possibly be relevant for subgroups of cancer patients [22]. A moderating effect was observed was for *alexithymia*, with low alexithymic EWI participants reporting larger reductions in number of telephone calls to their GP than low alexithymic women in the EWI group and controls. There are several reasons for suspecting a moderating role of alexithymia. One hypothesis states that individuals who have difficulties describing their emotions, or lack the skills and ability to reflect upon- and process their experiences, will benefit less from the emotional disclosure instructions of EWI [26, 27]. On the other hand, it could also be hypothesized that the structured and time-limited writing could be an opportunity for safe processing of traumatic material, giving alexithymic participants sufficient control over the disclosure process to allow themselves to be involved in the process. This latter hypothesis has found support in a study showing EWI to be more beneficial for healthy individuals with high scores on alexithymia [55], however, our findings appear to support the first.

The results also indicated that women who wrote about their cancer showed reduced healthcare utilization in the form of fewer GP contacts when compared to controls, while women who chose to write about other traumatic experiences did not. However, there was a baseline difference between groups both in terms of GP visits (p = 0.015) and phone calls (p = 0.036) with women writing about their own cancer scoring higher. One could speculate that those women who are more active health care users think more about their cancer experience and chose to write about it. For these women, EWI may be particularly helpful. Given the higher baseline level, it is, on the other hand, also possible that the moderation effect detected is an effect of regression toward the mean.

While previous EWI studies with cancer patients have constrained the participants to write about their cancer [22], participants in the majority of the non-cancer studies could select their own writing topics. The breast cancer could theoretically no longer be distressing or even be the most traumatic event for patients who have completed treatment, and when restricting patients to write about their cancer, some may thus be asked to write about a relatively non-traumatic event. To our knowledge, our study is the first to examine whether effects of EWI differ depending on the participants’ choice of writing topic, and we have no clear explanation for our results. It is possible that some individuals may have experienced writing about their own cancer as threatening and therefore chose to write about more superficial topics or less specific traumas, thereby perhaps reducing the possibility of an effect. Previously reported data from the present study [11] showed a similar pattern for psychological outcomes, with women writing about their cancer reporting fewer depressive symptoms and higher levels of positive mood post-intervention than controls, whereas no differences were found for women who wrote about other topics. It should be noted, however, that the interpretation of the results concerning GP visits and telephone contacts depends on the degree to which number of GP contacts can be interpreted as indicative of physical health. In some cases, reduced healthcare utilization could indicate insufficient and delayed response to symptoms, while in other cases, fewer contacts with the healthcare system could indicate a reduction in physiological arousal and reduced tendencies to notice and report physical symptoms.

We found no moderating effects for the remaining factors believed to be associated with emotion regulation: repressive coping style, rumination or social constraints. One comprehensive model of emotional non-expression describes five steps: 1) prereflective action, 2) conscious perception of the emotional response, 3) labeling and interpretation of the response, 4) evaluation the acceptability of the response, and 5) perceived social context for expression [56]. Repressive coping [24] can be viewed as failure at step 2, and repressive copers having difficulties acknowledging and identifying negative emotional experiences may benefit less from written emotional disclosure [26]. Research on EWI and repressive coping is, however, limited [26], and the validity of the available operational definitions of repressive coping style may be questionable [2, 25]. Social constraints, i.e., emotional inhibition due to inadequate opportunities to express emotions, are difficulties at step 5 [32], and are the most frequently researched moderator of EWI in cancer patients and survivors so far, with three studies finding benefits of EWI among participants reporting high social constraints [57–59]. Although the findings are inconsistent [60], social constraints remain a theoretically and empirically promising moderator to be studied further. In contrast to emotional non-expression constructs, rumination refers to excessive emotional expression which has been associated with intensified and prolonged negative emotions [28]. Although studies with healthy participants suggest that EWI buffers against maladaptive rumination [30, 31], rumination did not moderate the effect on physical health-related outcomes in women treated for breast cancer. One possibility is that rumination includes both adaptive and maladaptive factors [61].

The present population-based study is the largest RCT of EWI with cancer patients so far, enabling us to detect small effects. Second, we included a manipulation check revealing statistically significant and large effects (*d* = 0.84–1.04) in the expected direction. The effects were comparable to previously reported manipulation check outcomes [50, 57], and indicate that the intervention succeeded in influencing the women’s emotional responses. Some potential limitations could, however, have influenced the results. First, 44% of the eligible women actively declined or failed to respond to the invitation to participate. Second, 17% terminated their participation or failed to return follow-up questionnaires. Third, there are some issues concerning writing topic. One concern could be that we distinguished between women who wrote about their own cancer experience in any one of three sessions and women who wrote about another traumatic experience in all three sessions. Another concern is that we do not know the reasons for choosing not to write about cancer. For some women, the cancer might no longer be stressful, while for others the cancer might be experienced as too stressful to write about. As previously reported [11], participants writing about their cancer did not report greater cancer-related distress at baseline than those writing about other topics. Furthermore, lack of randomization may limit the generalizability of differences found between writing topic groups. A three-arm trial with a third group randomized to a group restricted to write about their cancer was considered but abandoned due to statistical power considerations. Finally, the women who agreed to participate could have been physically less affected by their cancer and cancer treatment than non-participants, leading to floor effects blurring effects of the intervention. It should be noted, however, that there were no disease, treatment, or sociodemographic differences between participants and dropouts and no baseline differences between EWI and CTRL participants.

## Conclusions

The results from our large randomized trial are concordant with previous findings suggesting that EWI is unlikely to be a generally applicable intervention to improve health-related outcomes in cancer patients and survivors. However, although effects were small, written disclosure could have a beneficial impact for a subgroup of individuals low in alexithymia and who write about their own cancer. Future EWI studies with cancer patients could focus on specifically testing the efficacy of various intervention characteristics, i.e., writing topic, in groups with specific theory-driven characteristics.
